# Trauma-Informed Healthcare Leadership? Evidence and opportunities from interviews with leaders during COVID-19

**DOI:** 10.1186/s12913-024-10946-9

**Published:** 2024-04-24

**Authors:** Sonia Rose Harris, Alexis Amano, Marcy Winget, Kelley M Skeff, Cati G Brown-Johnson

**Affiliations:** 1 School of Social Work, University of Minnesota, St. Paul, MN USA; 2 UCLA Fielding School of Public Health, Los Angeles, CA USA; 3 Stanford University School of Medicine, Palo Alto, CA USA

**Keywords:** COVID-19, Managers, Administrators, Leadership, Qualitative methods, Burnout

## Abstract

**Background:**

COVID-19 impacted the mental health of healthcare workers, who endured pressures as they provided care during a prolonged crisis. We aimed to explore whether and how a Trauma-Informed Care (TIC) approach was reflected in qualitative perspectives from healthcare leaders of their experience during COVID-19 (2020–2021).

**Methods:**

Semi-structured interviews with healthcare leaders from four institutions were conducted. Data analysis consisted of four stages informed by interpretative phenomenological analysis: 1) deductive coding using TIC assumptions, 2) inductive thematic analysis of coded excerpts, 3) keyword-in-context coding of full transcripts for 6 TIC principles with integration into prior inductive themes, and 4) interpretation of themes through 6 TIC principles (safety; trustworthiness and transparency; peer support; collaboration and mutuality; empowerment, voice, and choice; and awareness of cultural, historical, and gender issues).

**Results:**

The actions of leaders (*n* = 28) that were reported as successful and supportive responses to the COVID-19 pandemic or else missed opportunities reflected core principles of Trauma-Informed Care. To promote *safety*, leaders reported affirmative efforts to protect staff by providing appropriate physical protection, and enhanced psychological safety by providing channels for communication about emotional well-being. To promote *trustworthiness and transparency,* leaders listened to their staff, shared current COVID-19 information, and increased frequency of meetings to disseminate accurate information. To promote *mutual support*, strategies included wellness check-ins, sharing uplifting stories, affirming common goals, articulating fears, and leading by example. Examples of *empowerment* included: making time and adjusting modalities for flexible communication; naming challenges outside of the hospital; and functioning as a channel for complaints.

Reported missed opportunities included needing more dedicated time and space for healthcare employees to process emotions, failures in leadership managing their own anxiety, and needing better support for middle managers. Awareness of the TIC principle of *cultural, historical, and gender issues* was largely absent. Results informed the nascent Trauma-Informed Healthcare Leadership (TIHL) framework.

**Conclusions:**

We propose the Trauma-Informed Healthcare Leadership framework as a useful schema for action and analysis. This approach yields recommendations for healthcare leaders including creating designated spaces for emotional processing, and establishing consistent check-ins that reference personal and professional well-being.

**Supplementary Information:**

The online version contains supplementary material available at 10.1186/s12913-024-10946-9.

## Background

In the early years (2020–2021) of the COVID-19 pandemic, the incidence of depression and anxiety increased in the United States (US), and overall psychological well-being decreased [1–3]. The mental health of healthcare workers was particularly negatively impacted as they endured unrelenting challenges in providing patient care during the prolonged crisis [3–5]. Negative contributors to mental health for these healthcare workers included: feeling stressed and stretched too thin, concern about exposing family/network to illness, emotional and physical exhaustion, a lack of appropriate emotional support from colleagues and supervisors, and struggles with managing home responsibilities and isolation caused by work requirements [6]. In response to these negative impacts on healthcare personnel, the US Department of Health and Human Services issued recommendations to transform workplace culture, with goals to 1) empower through reduced administrative tasks, 2) eliminate punitive policies for seeking mental health care, and 3) prioritize social connection by increasing flexibility in scheduling and building peer- and team-based care models [7]. Healthcare personnel were still expected to continue performing their duties and deliver quality healthcare, often without time for recovery or restoration [6, 7].

Previous disaster management reports (e.g., after the 2003 Severe Acute Respiratory Syndrome (SARS) outbreak [8], 9/11 attack on the United States, and Hurricane Katrina in New Orleans) include lessons learned about the impacts of traumatic events on healthcare workers, such as need for resources that facilitate reflection on new stressors, for systems that mitigate the impact of interpersonal isolation, and for mental health resources without financial barriers for healthcare workers [9–11]. Analyses of the impact of COVID-19 on healthcare workers showed similar themes [3, 6, 7, 10], and emphasized the need for hospital- and health system preparedness [12].

Trauma-informed care (TIC) is considered a gold standard for effective care to support patient mental health [13–16]. The TIC framework emphasizes a shift from asking “what’s wrong with you” to “what happened to you,” emphasizing the need for a complete understanding of a person’s lived experience in accounting for and understanding their actions [17]. This perspective underscores the need to understand a person’s behavior in the context of their life experience [17]. In medical centers, the application of TIC involves changing organizational culture through policies and procedures and incorporating an understanding of the impact on trauma into routine care [18]. The US Substance Abuse and Mental Health Services Administration (SAMHSA) outlines four key assumptions (four R’s) in TIC [19]: 1) Realize the impact of trauma on individuals, families, and groups; 2) Recognize signs of trauma; 3) Respond by applying major TIC principles; 4) avoid Re-traumatization. In addition to these four assumptions, TIC is built on 6 principles [19]: safety; trustworthiness and transparency; peer support; collaboration and mutuality; empowerment, voice, and choice; and awareness of cultural, historical, and gender issues and oppression. TIC has been widely applied, for instance in schools, social work practices, treatment of domestic violence, juvenile justice, homelessness services, and child welfare agencies [15, 16, 18, 20, 21]. Within the last decade, TIC principles have also been applied in healthcare settings; reports in healthcare indicate that TIC can improve patient care outcomes [13, 22].

We aimed to explore whether and how a Trauma-Informed Care (TIC) approach was reflected in qualitative perspectives from healthcare leaders of their experience during COVID-19 (2020–2021). Our hope was that this analysis could inform leaders in healthcare about how to support teams and avoid pitfalls during ongoing and future crises.

## Methods

Participating Institutions and Sampling: Administrative and medical leaders at four healthcare organizations across the United States (from Colorado, Michigan, and Northern California) were invited to participate in a qualitative study. Eligibility included Vice Presidents, Directors, Division Chiefs, and other high-level leadership, with goal of a sample balanced across levels of leadership and also subject matter expertise (e.g., Nursing, IT, Finance.) Institutions represented a convenience sample including three academic medicine centers and one community institution.

Interview Questions/Guide: The interview guide (Additional File 1) was developed based on our team’s previous study [23], and focused on understanding healthcare leader feelings of joy and distress during COVID-19. The guide aimed to identify both gratifying and distressing professional experiences prior to and during the COVID-19 pandemic. We received pilot feedback from a high-level administrator with expertise in wellness.

Recruitment Strategy: Per methods outlined in Skeff et al. [23], we recruited in a multi-step process to protect the confidentiality of participants. Once a site contact was in place at each institution, the research team furnished them with IRB-approved introduction emails, which underscored research goals and purpose. These emails were sent to potential participants directly from the site contacts. Subsequently, the research team (AA/CBJ) responded directly to potential participants, without cc’ing connecting site contacts. We contacted potential participants no more than three times by email; site contacts were not informed as to who did or did not choose to participate.

Interviews: Two women researchers, with PhD and Master’s level training and expertise in qualitative research (CBJ, AA) conducted phone and video interviews (Zoom, Zoom Video Communications, San Jose, USA); Site A and B interviews were collected August to September 2021, Site C interviews October to November 2021, and Site D interviews in November 2021. Interviews were in-depth and semi-structured, each interview (one per participant) lasted between 30–60 min. One interviewer was additionally a subject matter expert in a related topic area: physician wellness and burnout (CBJ).

Participants provided written consent to participate and verbal consent to record interviews and were assured of the anonymity of interview reporting. Interviews were professionally transcribed (Rev, Rev.com, Inc, San Francisco, USA); any identifying information was deleted.

Qualitative analysis method and rigor: Data analysis for this study used an interpretative phenomenological analysis with theme clustering using TIC Framework [24]. Data analysis was conducted in four phases: 1) a priori coding using the four TIC assumptions; 2) inductive thematic coding of those excerpts; 3) keyword assisted coding of the full transcripts for elements of the six TIC Principles, and subsequent integration of those excerpts into prior inductive themes; and 4) interpretation of themes through the six TIC Principles. First, we used a priori coding with the deductive framework of TIC assumptions: realize, recognize, respond, and resist re-traumatization*.* Two qualitative researchers (AA and SRH) consecutively reviewed all transcripts and coded the excerpts to the four assumptions. We identified significant overlap between the four assumptions within the data set; multiple excerpts were coded for all four TIC assumptions. While this addressed our objective of *whether* TIC elements could be observed in leadership experiences of gratification and distress, it did not address our subsequent question of *how* TIC might be evidenced in interviews.

Thus, as a second phase, SRH and CBJ used consensus coding approaches to organize TIC excerpts into inductive themes (e.g., listening) using twice-weekly meetings to come to consensus through an iterative process. We simultaneously reviewed literature for other Trauma-Informed Care organizing frameworks to help understand the data.

A third phase of analysis involved mapping inductive themes from the TIC excerpts to the 6 principles of Trauma-Informed Care*:* 1. Safety; 2. Trustworthiness and transparency; 3. Collaboration and mutuality; 4. Peer support; 5. Empowerment, voice, and choice; and 6. Awareness of cultural, historical, and gender issues*.* Safety was defined as all people within the organization feeling physically and psychologically safe. Trustworthiness and transparency were defined as organizational activities conducted transparently with a goal of increasing trust. Peer support refers to mutual self-help. Collaboration and mutuality were defined as emphasizing teamwork and power sharing. Empowerment, voice, and choice refer to building on individual strengths and shared decision making and choice. Finally, awareness of cultural, historical, and gender issues was defined as awareness of oppression with efforts to move away from prejudice and bias. These definitions were adapted from the Substance Abuse and Mental Health Service Administration [19]. Two authors (SRH and CBJ) met weekly to come to consensus over a two-month period [25]. In addition to this focused consensus analysis, all co-authors reviewed developing organizational patterns of the results during monthly research team meetings.

To ensure a comprehensive account of healthcare leaders’ experiences with this trauma-informed lens, one researcher (SRH) conducted additional coding using a modified keyword-in context approach [26]. SRH used keywords to search the data set for TIC principles and close synonyms using qualitative software (NVivo 12, QSR International, Melbourne, AUS). Passages containing a keyword were reviewed by two authors (SRH and CBJ) and incorporated into the analysis when the passage included a term used in a way that matched the definitions of the TIC principles used in this dataset. Final structure for the data was organized by the TIC principles.

This project was presented for discussion and feedback in a lecture format and small group sessions to over 40 healthcare leaders interested in clinician wellness and healthcare worker burnout in February and March 2024. This project was approved by our Institutional Review Board (Protocol # 39948).

## Results

### Participants

Twenty-eight interviews were conducted via phone or Zoom with participants from four healthcare institutions across the United States (in Colorado, Michigan, and Northern California). These institutions represented both academic medical centers and community hospitals. Participants included healthcare leadership in finance, service lines, operations, and education. They filled specific roles of Chief Financial Officer, Chief Nursing Officer, Chief Medical Officer, Executive Director, or Director. Participants were 61% women (*n* = 17/28) and included ages across deciles from 30 to 70. 75% were white (*n* = 21/28) and all had 10 + years of experience in the field of health care administration. All participants who consented for an interview completed their interview. To maintain anonymity, participants have been assigned a random participant number (1–28). We use the term “leaders” throughout our results to highlight their role as leaders in their setting.

Overall, the actions of leaders that were reported as either successful responses to the COVID-19 pandemic or missed opportunities to alleviate distress mapped to core principles of trauma-informed care. Safety; trustworthiness and transparency; and empowerment, voice, and choice were reflected in our data. Principles of peer support and collaboration and mutuality were combined in our analysis as *“mutual support”* because while we saw examples of teamwork and power-sharing (collaboration and mutuality), there were not distinct elements of mutual self-help (peer support) perhaps due to power differentials between staff and leadership. The sixth assumption, awareness of cultural, historical, and gender issues, was largely absent from our data set. Leaders’ supportive actions and strategies are presented by TIC principle below. Table 1 provides further qualitative examples, organized first by TIC principle, then by theme. Table 1 also includes recommended future actions based on our data.

**Table 1:** Leaders’ actions mapped to trauma-informed principles including specific future actions leadership can take

TIC Principle	Themes	Exemplar Quote(s)	Recommended Action
**Safety**	Promoting physical protection	*“Even though you know that it’s not best practice, it’s better than nothing—but it’s not best practice….It was hard conversations about what is appropriate and what was the best we could do versus best practice.” (Participant 2)* *“In the end, we had absolutely zero negative patient outcomes, and our patients got better care. And the nursing staff was extremely satisfied with that, because they were able to not have to sit in that room with a patient shedding the virus.” (Participant 4)*	Increase communication to enhance trust and transparency when physical safety is a concern
	Not taking complaints personally	*“I’d get an angry email from the head of the department and he’s just looking… out for their staff right? And, and their nurses and their other employees. So I totally get it, right. Cause I’m doing the same for mine…” (Participant 8)*	Approach complaints from staff with empathy
	Missed Opportunity- Providing more attention, space, and time for emotional processing	*“Even now, we continue to see that and how people are very short with one another, and they don’t allow mistakes in other people and there’s a lot of unneeded hysteria, and I think that it stems back to us not really dealing with all of these emotions that we went through last year. And how do you deal with that on an administrative level, in a hospital when you have hundreds of people reporting to you? I don’t really know the answer to that, but if there was anything that I could have done better, that would’ve been anything.” (Participant 7*	Create safe spaces and when possible, give staff built in time for emotional processing which could include group talking spaces, time for exercise, nutritious food, or rest in the day
**Trustworthiness & Transparency**	Listening	*“I think that there's a value in just listening and acknowledging and we may not have an answer, and no one does. I feel that to recognize that it exists, I think goes such a long way and I think that's why we've developed such a great rapport with many of our frontline teams, because they feel and they see that we hear them.” (Participant 5)* *“So what helps… What I need to do is give myself time to listen and to let people know what we're doing for them. So, both those things take time… one kind of helps establish understanding, trust, and the other helps establish … the next level of trust where, ‘oh, he is acting on some of the things I expressed earlier.” (Participant 4)*	Listen without judgement to build trust and without immediate intention to solve
	Increasing frequency of information dissemination	*“When communication breaks down, that’s when rumor control starts. So, I think just being fully transparent with where you’re at, whether you had PPE, whether you didn’t. Whether you had to a process together to reuse N-95 s….[The staff] can trust you. Because it comes down to trust, it really does, it comes down to trust.* *Best practices I think is just to be open in your communications and transparent. Helping [staff] see that you're doing this for their best interests and the best interest of our patients and our colleagues. And we all want the same thing.” (Participant 25)* *“We made a decision as a command center group that the best that we could do would be to be visible and transparent, and really share… So, it was rough. I’m not going to lie. It was a rough time. But it was really about remaining calm, remaining focused, and being transparent, and being visible.” (Participant 13)* *“[Our department chair] would send almost daily email updates to the department faculty and staff, sharing the statistics, "This is how many patients we have on the inpatient unit. This is what the ER looks like," just different stories. "Here’s what’s just happening across [our organization] and the country." That was really helpful for us, to get that communication because even if we didn’t know what was going on, we knew that nobody else did either and nobody was holding things from us. We just didn’t know what was happening. So, he did a good job communicating.” (Participant 25)*	Foster transparency through increased information dissemination
	Making decisions transparently	*“I think also not always being able to answer questions or give our teams the most. Part of it is we don’t know the answers. And so not being able to provide direction to our team sometimes just because the information was changing. I felt a little bit not powerless, but I felt I always wanted to try to do more, and I couldn’t. So, I think that doesn’t feel great.” (Participant 18)*	Communicate to staff reasons for decisions and changes
	Missed opportunity- Overcommunicating on time off	*“When we have the command center set up and there’s these things going, and we say, 'Hey, it’s the weekend. You don’t need to do this.' And then yet on the weekend, we’re sending them texts or emails and all these pieces… I have definitely heard from quite a few of them for feeling like… They tell us to try and work on… taking care of ourselves and resting and removing ourselves. And then yet these emails come in and it definitely comes across as we expect you to respond to these.” (Participant 13)*	Be mindful and intentional with sending emails/other communications to staff on their time off. Think critically if it is essential communication
	Missed opportunity- Containing leadership anxiety	*“The major leadership failure here was that leaders didn’t contain anxiety within their teams. They let that anxiety fuel their own anxiety and threw it out for the organization. So we just all run and collided into each other. It was really damaging, and it took a lot of time to manage. So, how do you help people see, this is about my own like reflection, containment. What’s real, what’s anxiety. Even if I’m anxious, how do I contain my anxious? Because the world is scary right now… In order to be a learning culture. I think we have to be able to have enough insight to separate emotion from the reality or at least know.” (Participant 1)*	Leverage support of leadership peers to process personal anxiety as opposed to channeling frustration toward staff
**Empowerment**	Communicating with flexible times and modalities	*“I gave my personal cell phone out in addition to my work cell phone, so that the staff could text or call me at any point in time. And they did, and I answered them back and went down and listened to them and their fears…Very difficult time.” (Participant 4)*	Empower staff to choose best mode and time for communication
**Mutual support**	Checking on Wellness and Creating Personal Touch Points	*“We established this routine of almost good morning type emails to where… almost akin to if you were walking down the hallway and going in somebody’s door and just saying good morning. We increased the frequency of our manager touch-bases fourfold. It was good from a project perspective, but it was also good from a wellness perspective, just from the standpoint of 'we got to just talk'.” (Participant 22)* *“[The administration] introduced just a lot of conversations, a lot of time on just: how is everyone? What are people thinking about? What are you stressing about? What we learned is uncertainty [is a] cause of stress for people, right? We don’t know when we’ll be back at work, but… they knew that I had a plan.” (Participant 28)*	Proactive wellness check-ins prompted by leaders to establish mutual support
	Leading by example	*“I remember this one time my team doesn’t want to come in back to work, I have a patient I need to discharge. He’s homeless, he refused to wear a mask. He’s coming out to the nursing station, nursing staff, doctors freaked out. I remember, I wear a mask … At that time, we also didn’t have all the PPE that we have, right? I physically went into the room and I have to push the patient’s belongings out, so he can go to a hotel. Somebody has to do the job and at the end, someone has to do it. If nobody does it, a leader has to step in and do it.” (Participant 3)*	Model desired behavior of staff to enhance mutual support
	Identifying a common goal	*“We started to show up day in and day out together with the same group of people that for me, I was really new to the organization that I’m working at now, and so I did not have the long-lasting relationships as my peers had. Many of them grew up in this system together, and so I felt very fortunate that it was a very inclusive environment. Again, I think a lot of that had to do with that shared goal.” (Participant 5)* *“I think it was obviously a very scary time, a lot of uncertainty surrounding when the pandemic first became, I guess, an outbreak. And one of those things that I felt like went really well as a hospital administrator is the team got together very quickly. The common goal was definitely our motivator to be able to kind of think things through.” (Participant 5)*	Use a common goal to establish communal support, return to common goal as needed when facing challenges
	Sharing positive messages	*“What I really appreciated about our team was it was as though different people were at a high on different days. And so, those people who were feeling better did a really nice job of just building up the team and supporting us and helping us feel good. Some days, I was scared and anxious and wasn’t sure what to do next. And thankfully, somebody else would be having a better day that day and would have some sort of uplifting story or some way just to motivate us and feel better…” (Participant 27)* *“Supporting everybody who was in a different situation. Some people had babies at home, some people had elementary school kids that you can’t just sit in front of a computer and think they’re going to work. There was no two people experiencing the same environment or anxiety, whether it was work-related or not.” (Participant 28)* *“And so, the team really did a nice job supporting each other through that. Then it was nice because then the things that we shared, the positive moments, they would take back to their teams. And so, then that team got another uplifting moment. It worked out well. People really rose to the occasion and really supported each other.” (Participant 27)*	Leverage and encourage people who are having good days to lift up people who need more support
	Supporting middle management	*“I think the first thing that comes to my mind is trying to figure out the best way to support middle management. Those people that are sort of in between trying to keep the team of frontline people together, but then also have the demands coming down from them, and trying to figure out with the current situation, how do we support that group, so they don’t feel like they’re supposed to be working 24 h a day, seven days a week, and help them address their own burnout.”(Participant 13)* *“I think that’s been a big struggle from our standpoint. We have seen more turnover in that area recently than we really would like to see. When I go out and around and talk with frontline staff, many of them say, 'I stay because of my manager.' Not because of senior leaders, they stay because of the person they have that interaction with. As a result, if we have those people leaving, then we have more potential for losing some of our frontline staff, which at this point we just simply can’t afford.” (Participant 13)*	Spend time building structures to support middle management including trauma-informed trainings to protect against burnout
**Cultural, Historical and Gender issues**	Naming Challenges Outside of the Hospital	*“And I think the one that was really scary, but I felt like I had to do it, was to just talk about race. … I could not let that moment in time go by us without acknowledging how much pain many people… were feeling.” (Participant 15)*	Engage with challenges occurring outside the hospital to empower staff to raise concerns and recognize depth of each person

I. ***Safety***. Healthcare leaders employed the principle of safety, which is defined as all members of the organization feeling both physically and psychologically safe, by (1) promoting protection to ensure physical health and (2) by not taking complaints personally. Leaders reported a missed opportunity for supporting psychological safety by not providing more attention, space, and time for emotional processing.
  1. **Promoting physical protection**. Healthcare leaders reported that addressing staff concerns about physical safety—including the limited supply of personal protective equipment (PPE)—was paramount to supporting staff wellness during COVID-19. Some department’s PPE allotment was lower than staff needs. The disparity in resources raised staff fears and anxiety. In response, leaders reported feeling successful when they increased communication and specifically identified best practices vs. realistic approaches in the current environment.
    Even though you know that [reusing masks with different patients, washing masks] is not best practice, it’s better than nothing—but it’s not best practice…. It was hard conversations about what is appropriate and what was the best we could do versus best practice. (Participant 2)
    One leader reported using extension intravenous (IV) tubing so that nursing staff could monitor the IVs of patients from outside the intensive care unit (ICU) room. While the participant reported receiving complaints from system executives, there were no negative patient outcomes and staff were extremely satisfied.
    *In the end, we had absolutely zero negative patient outcomes, and our patients got better care. And the nursing staff was extremely satisfied with that, because they were able to not have to sit in that room with a patient shedding the virus. (Participant 4)*
  2. **Not taking complaints personally**. In best-case scenarios, interviewed healthcare leaders managed to not take complaints personally or respond in anger when they received complaints from staff for situations beyond leadership control. This spirit of acceptance created space for venting frustration without retaliation, supporting psychological safety. When leaders found themselves the target of anger, effective managers worked to let it go. “The anger of the staff that’s directed towards you, even though it’s probably not personal, but you’re the punching bag about not keeping [staff] safe” *(Participant 1). *In best circumstances leaders looked for potential root causes of the frustration: fear, concern for their team, lack of control:
    *I’d get an angry email from the head of the department and he’s just looking… out for their staff right? And, and their nurses and their other employees. So I totally get it, right. Cause I’m doing the same for mine… (Participant 8)*
  3. **Missed opportunity- Not providing more attention, space, and time for emotional processing**. Interviewed healthcare leaders reported that they recognized a responsibility as team leads to create space for staff to process emotions by encouraging staff to speak freely without concerns of minimization or retaliation. They reported that not dealing with the emotions of staff during the crisis setting of COVID-19 had a lasting impact:
    *Even now, we continue to see that and how people are very short with one another, and they don’t allow mistakes in other people and there’s a lot of unneeded hysteria, and I think that it stems back to us not really dealing with all of these emotions that we went through last year. And how do you deal with that on an administrative level, in a hospital when you have hundreds of people reporting to you? I don’t really know the answer to that, but if there was anything that I could have done better, that would’ve been anything. (Participant 7)*
II. ***Trustworthiness and Transparency***. To promote trustworthiness and transparency, defined as organizational activities conducted transparently to increase trust, leaders reported: (1) listening; (2) increasing frequency of information dissemination; and (3) making decisions transparently. Missed opportunities to establish trustworthiness and transparency included: (4) overcommunicating on time off and (5) not containing leadership anxiety.
  1. **Listening**. Intentional listening in one-on-one interactions worked well to identify specific concerns. Leaders recognized their responsibility to “hear people out, because they need to feel like they've been heard” (Participant 4). Listening with patience established two levels of trust: “So what helps... What I need to do is give myself time to listen and to let people know what we're doing for them. So, both those things take time… one kind of helps establish understanding, trust, and the other helps establish … the next level of trust where, ‘oh, he is acting on some of the things I expressed earlier'” (Participant 4). By listening, one participant learned that staff valued hearing that managers may not have an answer but were making efforts to address staff concerns:
    *I think that there's a value in just listening and acknowledging and we may not have an answer, and no one does. I feel that to recognize that [transparency] exists, I think goes such a long way and I think that's why we've developed such a great rapport with many of our frontline teams, because they feel, and they see that we hear them. (Participant 5)*
  2. **Increasing frequency of information dissemination.** Healthcare leaders increased the frequency and modality of information dissemination about COVID-19 in response to the changing environment. Specific examples included: increasing team meetings; weekly huddles; and frequent actionable information-sharing. Leaders increased the frequency of communication to address the constant flux of COVID-19 information. A participant recalled increasing meetings to twice a month. Frequent communication in established settings created opportunities to address changing circumstances.
    Leaders recognized that timely sharing of information about decision-making and the availability of resources increased trust among staff members and inhibited the spread of rumors.
    *When communication breaks down, that’s when rumor control starts. So, I think just being fully transparent with where you’re at, whether you had PPE, whether you didn’t. Whether you had to a process together to reuse N-95s…. [The staff] can trust you. Because it comes down to trust, it really does, it comes down to trust. (Participant 25)*
  3. **Making decisions transparently**. When leaders were transparent about their decision-making processes, they could communicate effectively that they were acting in the best interest of staff and patients. When they lacked information due to changing guidelines or circumstances, they reported discomfort.
    *I think also not always being able to answer questions or give our teams the most. Part of it is we don’t know the answers. And so not being able to provide direction to our team sometimes just because the information was changing. I felt a little bit not powerless, but I felt I always wanted to try to do more, and I couldn’t. So, I think that doesn’t feel great. (Participant 18)*
  4. **Missed Opportunity- Overcommunicating on time off.** Healthcare leaders noted that too much communication, specifically on weekends, sent a message that staff should continue to work even on their days off, which could undermine trust.
    *When we have the command center set up and there’s these things going, and we say, "Hey, it’s the weekend. You don’t need to do this." And then yet on the weekend, we’re sending them texts or emails and all these pieces... I have definitely heard from quite a few of them for feeling like... They tell us to try and work on... taking care of ourselves and resting and removing ourselves. And then yet these emails come in and it definitely comes across as we expect you to respond to these. (Participant 13)*
  5. **Missed Opportunity- Containing leadership anxiety**. Leaders reported missed opportunities that centered on failures to manage their own anxiety and contain it within their leadership team. Interviewees shared that they failed to protect staff from leadership anxiety, which created more chaos:
    *The major leadership failure here was that leaders didn’t contain anxiety within their teams. They let that anxiety fuel their own anxiety and threw it out for the organization. So we just all run and collided into each other. It was really damaging, and it took a lot of time to manage… (Participant 17)*
III. ***Empowerment, Voice, and Choice (Empowerment)***. Leaders communicated with staff with flexible times and modalities to promote individual empowerment defined as sharing decision making and choice. Leaders reported that they gave staff options for how and when to connect. Building pathways of support required leaders “to flex our hours, be on our emails constantly, circle back with the staff” (Participant 4). Leaders also added communication modalities - frequent emails, in-person and virtual huddle meetings, personal cell phone communications, texting options, and in-person meetings - to increase accessibility:
  *I gave my personal cell phone out in addition to my work cell phone, so that the staff could text or call me at any point in time. And they did, and I answered them back and went down and listened to them and their fears…Very difficult time. (Participant 4)*
IV. ***Mutual Support***. To promote mutual support, defined as mutual self-help and teamwork, leaders reported: (1) checking on staff wellness and creating personal touch points to foster connection, particularly in virtual settings; (2) leading by example; and (3) sharing positive messages. Leaders reported a missed opportunity for mutual support by not supporting middle managers.
  1. **Checking on wellness and creating personal touch points**. Leaders strengthened individual relationships with staff through developing or expanding wellness check-ins and personal touch points. These conversations set precedents to communicate about emotional well-being. For example, one leader shared that, in response to work-from-home requirements, they transitioned to “good morning” (Participant 22) emails, increased touch points with staff, and articulated interest in the impact of (home) stressors outside of work. These efforts opened pathways of communication and supported staff well-being:
    *We established this routine of almost good morning type emails... almost akin to if you were walking down the hallway and going in somebody’s door and just saying good morning. …it was also good from a wellness perspective, just from the standpoint of “we got to just talk”. (Participant 22)*
  2. **Leading by Example***.* Leaders led by example, modeling desired behaviors while working to understand the unique needs of staff members. One leader noted that it was the responsibility of the leader to step in when no one else would:
    *I remember this one time my team doesn’t want to come in back to work, I have a patient I need to discharge. He’s homeless, he refused to wear a mask. He’s coming out to the nursing station, nursing staff [and] doctors freaked out. I remember, I wear a mask ... At that time, we also didn’t have all the PPE that we have, right? I physically went into the room and I have to push the patient’s belongings out, so he can go to a hotel. Somebody has to do the job and at the end, someone has to do it. If nobody does it, a leader has to step in and do it. (Participant 3)*
  3. **Identifying a common goal**. Identifying a common goal motivated teams and set universal expectations for how to respond to the new environment. One participant reported that a shared goal equipped teams to respond cohesively to the crisis.
    *I think it was obviously a very scary time, a lot of uncertainty surrounding when the pandemic first became, I guess, an outbreak. And one of those things that I felt like went really well as a hospital administrator is the team got together very quickly. The common goal was definitely our motivator to be able to kind of think things through. (Participant 5)*
    One leader spoke specifically about the common goal of “getting the job done” (Participant 5)*. *This participant saw team members pivot from their own responsibilities to working collaboratively for team success.
  4. **Sharing positive messages.** Healthcare leaders reported that positive messages motivated the team and alleviated stress and fear during the early crisis of COVID-19. They reflected awareness that the challenges would impact each person differently recognizing that individual circumstances and prior experiences influence reactions, articulating both the *recognize *and *realize *assumption in TIC. In times of crisis, people will experience highs and lows at different times. The responsibility to engage in uplifting communication shifted by day and mood among team members. Positive messages served to “motivate” and to help people “feel better” (Participant 25).
    Additionally, sharing positive moments and uplifting stories helped people “rise to the occasion” (Participant 25). To boost morale and positive messaging, leaders focused team messages to emphasize that no two people were having identical experiences during the pandemic, whether at work or at home.
    *Supporting everybody who was in a different situation. Some people had babies at home, some people had elementary school kids that you can’t just sit in front of a computer and think they’re going to work. There was no two people experiencing the same environment or anxiety, whether it was work-related or not. (Participant 28)*
    Positive communication unified and strengthened teams to respond to the crisis:
    *What I really appreciated about our team was it was as though different people were at a high on different days. And so, those people who were feeling better did a really nice job of just building up the team and supporting us and helping us feel good. (Participant 22)*
    Even in a shared experience, people react differently; however, leaders emphasized unequivocal support for the team, regardless of where they might be.
  5. **Missed Opportunity- Supporting middle management**. Finally, one leader noted that the demands on middle management coming from front-line teams as well as executive and higher-level leadership were extreme. These leaders could have been better supported by higher-level leadership.
    *I think the first thing that comes to my mind is trying to figure out the best way to support middle management. Those people that are sort of in between trying to keep the team of frontline people together, but then also have the demands coming down from them, and trying to figure out with the current situation, how do we support that group, so they don’t feel like they’re supposed to be working 24 hours a day, seven days a week, and help them address their own burnout. (Participant 13)*
V. ***Cultural, Historical, and Gender Issues***. One leader reported naming and addressing oppression and racism occurring outside the hospital, acknowledging that there would be an impact on staff. As team leads, participants were also responsible for addressing challenges that could impact staff function, even when they occurred outside of the hospital system and outside of leadership control. For example, one leader described using their leadership platform to talk about George Floyd’s murder and the potential impact of racism on staff:
  *And I think the one that was really scary, but I felt like I had to do it, was to just talk about race. … I could not let that moment in time go by us without acknowledging how much pain many people… were feeling. (Participant 15)*

### The Trauma-Informed Healthcare Leadership approach

**Fig. 1:**
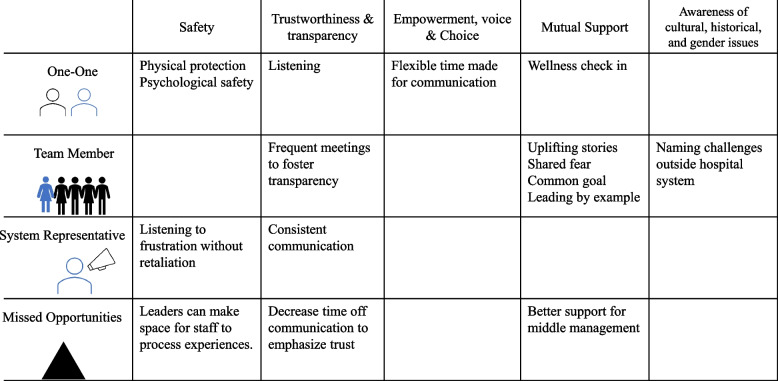
Trauma-Informed Healthcare Leadership: Successful actions and missed opportunities by leaders in one-on-one, team, and system relationships during COVID, mapped to principles of Trauma-Informed Care

Our reported results informed the nascent Trauma-Informed Healthcare Leadership (TIHL) approach. The TIHL approach (Fig. 1 & Table 2) highlights successful actions and missed opportunities by leaders in different relationships/settings: one-on-one management, team participation and leadership, and at the system level. To promote *safety*, leaders reported affirmative efforts to protect staff by providing appropriate physical protection (e.g., PPE), and enhanced psychological safety by providing channels for communication about emotional well-being. To promote *trustworthiness and transparency,* leaders listened to their staff, shared up to date COVID-19 information, and increased frequency of huddles and meetings to disseminate accurate information. To promote *mutual support*, strategies included wellness check-ins, sharing uplifting stories, affirming common goals, articulating fears; and leading by example. Examples of *empowerment* included: making time and adjusting modalities for flexible communication; naming challenges outside of the hospital; and functioning as a channel for complaints.

**Table 2:** Actions within the Trauma-Informed Healthcare Leadership framework, per relationship level

**One-on One Leadership**
Checking on wellness and creating personal touch points (*Mutual Support, Empowerment)* Providing opportunities for emotional processing in a way that best supports the individual in that moment (i.e. exercise, talking through emotions, rest) (*Mutual Support, Safety)* Listening completely without judgement (*Safety, Mutual support)*
**Team Participation and Leadership**
Identifying common goals ahead of time, while recognizing goals might change (*Mutual Support)*
Acknowledging that different people will experience highs and lows at different times; leverage the highs (*Mutual Support)*
Creating pathways for teams to communicate positives to uplift the team (*Mutual Support)*
Ensuring that actions leaders ask teams to do are ones they would be willing to do themselves; leading by example (*Trust and Transparency, Safety)*
In times of crisis, instituting more checkpoints and detailed pathways for communication (*Trust and Transparency, Safety)*
Meeting with teams frequently to disseminate information (*Trust and Transparency)*
Naming challenges inside and outside the system (*Oppression, Safety)* Training to support middle managers (*Mutual Support)*
**System Level**
Adopting a mechanism to surface complaints that is not retaliatory (*Trust and Transparency, Safety)* Building an inclusive culture through a reparative approach such as the Healing ARC (accountability, redress, closure) framework or restorative justice circles (*Culture, Gender and Oppression)*

## Discussion

This study documents evidence of hospital administrator actions reflecting principles of trauma-informed care (TIC) during the crisis setting of the COVID-19 pandemic. Leaders reported successful actions and missed opportunities to support staff that mapped to core principles of trauma-informed care: safety; trustworthiness and transparency; peer support/collaboration and mutuality; and empowerment, voice, and choice. Awareness of the TIC principle of cultural, historical, and gender issues was largely absent.

### The Trauma-Informed Healthcare Leadership approach

Traumatic events, whether experienced at work or outside of employment, can manifest in behaviors or feelings that may not be related to the original stressor [27]. Through this work, we identified core behaviors to seed the proposed Trauma-Informed Healthcare Leadership (TIHL) framework (Fig. 1 and Table 2). These behaviors, which are mapped to the 6 key principles of TIC, suggest actions leaders can take to prevent distress and/or mitigate trauma at different levels of relationships. Based on our data and evidence in the literature, we believe that the application of the Trauma-Informed Healthcare Leadership approach may establish some of the necessary supports to alleviate stress and anxiety related to traumatic events in healthcare—that can manifest as other behaviors or contribute to burnout. Like the Sanctuary Model or other trauma-informed organizational practices [28–31], the proposed Trauma-Informed Healthcare Leadership framework could support leaders and staff during healthcare system strain. The Trauma-Informed Healthcare Leadership framework, however, might be more accessible to a broader set of healthcare systems due to its concise approach and strong origin in the well-established Trauma-Informed Care principles. Perhaps a best-case scenario for a system interested in addressing healthcare worker needs during and after crisis would be to start with the Trauma-Informed Healthcare Leadership approach and extend into the Sanctuary Model as time and resources allow [28].

### Addressing Gaps: Supporting mental health, providing dedicated time and space for emotions, elevating needs of middle managers

Notably, leaders reported missed opportunities in the areas of *safety, trust, *and* mutual support*, pointing to an underlying gap in leadership awareness and tools to support health care worker emotional experience and mental health. Leaders noted a range of failures related to mental health supports, from a lack of dedicated space for healthcare employees to process emotions related to stressors, to limitations in managing leadership anxiety. Successes exist in the behavioral health sphere in the form of trauma-informed supervision, which we posit could be applied to remedy these gaps. Behavioral health supervision works to prevent and mitigate vicarious trauma and serves as a protective factor against anxiety and depression [14]. This practice combines knowledge of trauma with supervision and emphasizes that the relationship between supervisor and supervisees must be built on trust, with clear expectations regarding boundaries, listening, and the open exchange of feedback [14]. Additionally, emotional processing may look different for each individual and change on any given day [32]. Creating space for emotional processing could be designated spaces for verbal communication about feelings or creating time in the day for rest, exercise, or preparing nutritious food [32].

While trauma-informed supervision is useful to address gaps, it manifests in a one-to-one relationship; further research is needed to address the application of trauma-informed supervision in a one-to-many or system-mediated relationship. To support teams of health care workers, individuals must have access to a variety of psychological supports—not only through resources such as Employee Assistance Programs, but also by the allocation of time and space for individual processing, as well as with their administrators and managers. Previous literature indicates that providing dedicated spaces through restorative circles (i.e., structured times and space to discuss emotions and perception) to process emotions and self-reflect could increase self-awareness, compassion, and tolerance of stress for all involved.[33]

Lastly, with respect to leadership-identified gaps, this study also validates the need for targeted support for middle management and frontline managers. Middle managers are essential to the success of the healthcare system because these leaders provide direct support to staff [34], and this direct manager relationship is more predictive of retention than any other relationship in a work environment [35–37]. Additionally, previous work indicates that leadership qualities of supervisors impact staff well-being and predict burnout [38]. However, despite their importance, middle and front line managers may be limited by HR and system constraints [39, 40]. Therefore, leveraging resources to support middle level managers may enhance both safety and mutual support (TIC Principles)—and be protective against burnout and attrition. Application of trauma-informed care in elementary school settings that trained administrators in trauma-informed approaches (to apply to students) reduced administrator burnout [41]. It stands to reason that trauma-informed training for leadership and middle managers with the first goal of supporting their staff may similarly have additional benefits of reducing leadership burnout and fatigue.

### Addressing (Lack of) Awareness of Cultural, Historical, and Gender Issues

To address observed blind spot of *cultural, historical, and gender issues***,** system level anti-oppression work is needed. Responses that mapped to the final TIC principle, awareness of *cultural, historical, and gender issues***,** were very rarely discussed by our participants, suggesting that leaders are further hindered in their ability to address the intersection of traumatic experiences. Historically marginalized healthcare workers may be at greater risk for burnout at work[42, 43] and poorer mental health in general [44], in addition to potentially being more vulnerable, as a population, to the impacts of trauma [45, 46]. Previous research on racism experienced by physicians of color in health-care settings reports that physicians frequently face overt racism as well as microaggressions in their workplace [47].

Creating recognition of societal, community, and organizational oppression among healthcare leaders and staff may be the first step in addressing *cultural, historical, and gender issues*, but naming these issues is only the very first step. Healthcare system leadership may look to anti-racist actions in healthcare delivery to address racism and oppression in the hospital system, such as The Healing ARC or Presence 5 for Racial Justice [48, 49]. The Healing ARC, a race-conscious approach developed by two physicians at Brigham and Women’s Hospital, calls for a shift towards holding healthcare institutions accountable for actions that result in racial inequities in health [50–52]. The Healing ARC is built on three components: 1) acknowledgement, meaning acknowledging how racism has added to inequities in health; 2) redress, meaning putting in place compensatory actions to account for actions; and 3) closure, meaning institutions work collaboratively with the community that has been harmed to affirm that harm has been addressed and repaid [50–52]. These components are similar to other restorative justice approaches emphasizing acknowledgement of harm [33, 48]. Leaders can look to establish this or a similar model within their own system to address institutionalized racism and oppression [33, 53].

#### Limitations

This study has limitations in terms of data collection and analysis. First, the focus of the interviews was not trauma-informed care. Had we asked about TIC principles, participants may have provided more focused responses, particularly regarding awareness of oppression. However, the open nature of interviews can also be seen as a strength of this study. Additionally, we only conducted one interview with each participant. Given the sensitive nature (asking about moments of joy and distress and role during the pandemic) of our interviews, a longitudinal approach may have supported greater relationship rapport and trust, which could have provided more robust examples. Finally, we cannot speak to motivation of behaviors, i.e., were their responses intentionally or unintentionally brought out of trauma-informed care. In terms of participants, our sample only includes leadership. To strengthen the Trauma-Informed Healthcare Leadership approach, it will need validation across staff. Finally, trauma-informed care does not inform a strict adoptions model; instead, this approach offers principles and assumptions. Adapting to TIHL will include defining TIC in the healthcare leadership space while offering recommendations. We hope this work is a first step in that direction.

## Conclusions

Healthcare leaders demonstrated application of principles of trauma-informed care during the early crisis of COVID-19, specifically supporting safety, trust, mutual support, and empowerment for staff. They also reported gaps and missed opportunities related to: 1) providing more dedicated time and space for healthcare employees to process emotions related to stressors, 2) failures in managing leadership anxiety, and 3) the need for better support for middle managers. Notably, few participants addressed the principle of cultural, historical, and gender issues, indicating that efforts to promote anti-racist and anti-oppression inclusive work cultures that actively work to address continued discrimination and oppressive practices may be needed. Based on our results and corroborating literature, we propose the Trauma-Informed Healthcare Leadership framework, a straightforward approach with specific recommendations for leadership. Future research can validate and query this emerging approach and expand to middle managers and non-administrator leaders within the healthcare system.

### Supplementary Information

**Additional file 1.** Interview Protocol

## Data Availability

The data that supports these findings are available from the corresponding author, CBJ, upon reasonable request.

## Abbreviations

TIC: Trauma-Informed Care
TIHL: Trauma-Informed Healthcare Leadership
US: United States
SARS: Severe Acute Respiratory Syndrome
SAMHSA: Substance Abuse and Mental Health Services Administration
IRB: Institutional Review Board
PPE: Personal protective equipment
IV: Intravenous
ICU: Intensive care unit
