# Supplementary material for: Trauma-Informed Healthcare Leadership? Evidence and opportunities from interviews with leaders during COVID-19
Source: BMC Health Serv Res. 2024 Apr 24;24:515. doi: 10.1186/s12913-024-10946-9 (PMC11044408; doi:10.1186/s12913-024-10946-9)
Supplement: Supplementary file 1 — Additional file 1. Interview Protocol [file 12913_2024_10946_MOESM1_ESM.docx]

**Administrator Interview Guide**

*These questions are designed to be mainly used as open-ended questions around each of the major areas. Specific questions within each content area are to be prompts by the interviewer of the individual physicians.

**INTRODUCTION**

Hi, my name is XXXXX and I am a part of a Stanford School of Medicine research and quality improvement group called the Evaluation Sciences. Before we begin, I will be reading through an oral consent script that will explain this study and describe what your participation entails.

You are invited to participate in an improvement research study to understand administrator experience as well as to ascertain administrator perceptions on past study findings concerning physicians’ experience with the Electronic Medical record. You will be asked to partake in an interview to answer questions about your experience as an administrator and about your thoughts on our past study findings.

Your participation will take approximately 30 minutes.

The risks associated with the study are minimal. The only risk is a breach of confidentiality.

We cannot and do not guarantee or promise that you will receive any benefits from this study.

You will receive not receive payment for your participation.

Please understand **your participation is voluntary** and you have the right to withdraw your consent or **discontinue participation at any time without penalty**. You have the right to refuse to answer particular questions. Your individual privacy and **confidentiality** of the information you provide will be maintained in all published and written data resulting from the study.

Your private information collected as part of the research, even if identifiers are removed, will not be used or distributed for future research studies.

**FOR QUESTIONS ABOUT THE STUDY**

*Questions:*  If you have any questions, concerns or complaints about this research study, its procedures, risks and benefits, you should contact the Protocol Director, Cati Brown-Johnson at (650)736-3394.

*Independent Contact:*  If you are not satisfied with how this study is being conducted, or if you have any concerns, complaints, or general questions about research or your rights as a participant, please contact the Stanford Institutional Review Board (IRB) to speak to someone independent of the research team at (650)-723-2480 or toll free at 1-866-680-2906. You can also write to the Stanford IRB, Stanford University, 1705 El Camino Real, Palo Alto, CA 94306.

With your permission, the interview will be recorded and transcribed so that the answers can be analyzed. All names and other identifiers will be removed from the transcriptions. The audio files will be destroyed in two years at the end of the study. The transcriptions will be stored until the HIPAA expiration date.

I would like to audio record the interview to help me focus on you instead of taking notes. Are you comfortable with me **audio recording** our confidential discussion?

- Ok, I am **turning on the recorder** now. Today is DATE.
- Can you **please say your name** and that you **consent to being recorded**?

**Positive Aspects of Administrator Role**

Let’s start off with positive aspects of your role.

1. **Do you refer to yourself as a healthcare administrator? How do you identify?**
2. **Broadly, what do you like about being a healthcare administrator?**
   1. What are the positive aspects of your role?
   2. What experiences in your day-to-day role bring you joy?
   3. Are their aspects of your role that bring you fulfillment?
   4. How did you end up here?

**Experience of Administrators**

We are interested in your biggest sources of fulfillment and stressors.

1. **Tell us a story about something good or that you felt really good about in your role as an administrator during the last year.**
   1. Tell us more about your emotions during this event.
   2. What triggered those emotions – please be as specific as possible.
2. **Tell us a story about something not so great in your role as an administrator during the last year.**
   1. Tell us more about your emotions during this event.
   2. What triggered those emotions – please be as specific as possible.
   3. Why did you have to do this activity? Who/what required it?
   4. Why do you think administrators, rather than others, are asked to do this activity?
   5. Who else could have completed this activity?
   6. Do you think this challenge was in place before the pandemic?
   7. Do you think that the challenges that were in place pre-pandemic have been amplified?
   8. Have you been asked to reflect on your experience during COVID? Why do you think that is?
   9. If yes, how did that make you feel?

**Feedback**

Communication between administrators and providers can be challenging.

1. **What are things that you do that work well and what doesn’t work well in communicating and giving feedback to providers?**

**Learning Culture**

We are interested in what believe are factors needed for an effective organizational structure.

1. **Have you faced a mistake as an institution? Could you describe what happened and what you think should happen.**
   1. What do you think are important variables for making an organization work?
   2. Where do you see the needs for effective administration and contributions of the team members? *(Does the organization care for contributions of team?)*
   3. If facing a mistake, how do you think it should be handled? (*How does the organization respond to mistakes?)*

**Results/Utility of Data on EMR Use and Physician Distress**

Our recent study found that physicians often compromise patient care, professional behaviors, and values in the process of completing EMR-related tasks.

**** Skip to Question 9 if 3 interviews have been conducted at a site*

1. **We wonder whether it could help administrators to know the compromises that physicians feel they are making?**
   1. Would that information help you? If so, what might you do?
   2. Are you surprised to hear this?
   3. Physicians stated that they often would not tell anyone about these compromises. Any thoughts regarding why they would be hesitant?
2. **Is there anything you want to do differently knowing that physician professional behaviors and values are being compromised?**
   1. How much control do you feel like you have to change things?
   2. Thinking about the future, how do you think administrators should ideally respond?
   3. Do administrators have similar compromises or hesitancies to share?
3. *** ONLY TO BE ASKED AFTER 3 INTERVIEWS AT A SITE: In thinking about communication with physicians, we heard in a past study from physicians that they may face challenges communicating distress concerning EMR use.

Let me read something to you, we heard from physicians in a past study.

[/Box/EMR Physician Distress-Skeff/Administrator Experience/Administrator Interview_EMR Excerpts.pptx](https://stanfordmedicine.box.com/s/sru8komp57eeqccov5cxyd9z3gc3ok1m)

- 1. How do you feel after hearing these excerpts? What are your first thoughts?
  2. Does hearing these passages impact your feelings about the issue?
  3. How would you handle this situation?
  4. Do you feel any differently than you did after we described the issue?
  5. How would you use this information? Would having access to detailed qualitative descriptions of institutional problems be useful to you as an administrator?
     1. If yes, how so?

**Wrap-Up**

1. Before we wrap up, is there anything you would like to add?
2. Are there any questions we should have asked?

**Just a few demographic questions before we wrap up:**

**What is your gender?**

**What is your age in decades?**

□ 20-29 □30-39 □40-49 □50-59 □60-69 □70-79 □Over 80

**What race and ethnicities do you identify with? You can pick as many as that apply:**

**How many years have you been in this profession?** □ <1 year □1-4 □5-9 □10+ years

**How many years have you been at your institution?** □ <1 year □1-4 □5-9 □10+
